# Current and future of targeted therapies against BCR::ABL kinases

**DOI:** 10.1186/s43046-025-00263-5

**Published:** 2025-04-07

**Authors:** Sridhar Jayavel, Manasvini Subramanian, Pradeep Kumar Kesavan, Suresh Jayavel

**Affiliations:** Madurai Kamaraj University, Madurai, India

**Keywords:** Chronic myeloid leukemia, Philadelphia chromosome, Tyrosine kinase inhibitors, BCR::ABL variant

## Abstract

Chronic myeloid leukemia (CML) is a kind of leukemia that arises due to the translocation betwixt chromosomes 9 and 22. Philadelphia chromosome is characterized by the BCR::ABL fusion gene, which results from this recombination. It transcribes into active tyrosine kinase variants such as P185, P190, P210, and P230, depending on breakpoint chain variations. The fusion protein, encodes tyrosine kinases with varying exons, resulting in uncontrollable ATP-utilizing downstream signaling activities. Targeted therapy with various tyrosine kinase inhibitors (TKIs) is used to combat BCR::ABL fusion kinases and increase the survival rate of patients. However, the incidence of TKI resistance among CML patients is widely noticed around the world. Hence, an elaborate and accurate understanding of the structural interactions between BCR::ABL encoded tyrosine kinases, which are responsible for sensitivity and resistance, is mandatory for hassle-free targeted therapy. This review is intended to cover the reported structural interactions between BCR::ABL variants and TKI ligands in detail to highlight strategies that may be applied in the near future to overcome the resistance and other cross-reactions.

## Introduction

Chronic myeloid leukemia (CML) is a type of blood cancer that impacts the bone marrow. It originates from a single abnormal cell that acquires the ability to proliferate uncontrollably due to the disorder in the hematopoietic stem cells. CML is reported to affect around 15% of all adult leukemia cases [1]. The presence of the Philadelphia chromosome is recognized as the primary causative factor in 95% of CML patients [2].

The global incidence of CML ranges from 0.4 to 1.75 per 100,000 individuals every year. This incidence varies across regions, with higher frequency reported in North America and Europe compared to Asia and Africa [3]. Age plays a crucial factor in the prevalence of CML, as the incidence of CML is notably higher among older individuals [4]. A cascade of events initiated by translocation in hematopoietic stem cells causes the transformation of CML, leading to the formation of the fusion gene. It is also important to note that the BCR::ABL gene is not an inheritable gene, and the transcription of the domains of the gene generates a chimeric protein with increased tyrosine kinase activity [5]. The SH3 domain regulates ABL kinase activity in normal cells and its deletion has resulted in uncontrolled cell proliferation. The constitutive tyrosine kinase activation in CML instigates the deregulation of several signaling pathways, like the Ras/MAPK and PI3K/Akt pathways [6]. These altered pathways enhance cell survival, proliferation, and resistance to apoptosis, thereby exacerbating uncontrolled cell proliferation [7].

CML typically advances through three phases: chronic, accelerated, and blast crisis [8]. Symptoms of the chronic phase experienced by individuals include tiredness, sudden weight loss, and enlargement of the spleen. Subsequently, the disease progresses to the formation of abnormal cells, and a decrease in the effectiveness of the treatment, leading to the accelerated phase. Further, the condition is distinguished by a notable rise in the percentage of immature blast cells, marking the blast phase. This progression often manifests with symptoms such as bone pain, hemophilia, and increased susceptibility to infection. Genetic instability among CML cells contributes to additional chromosomal abnormalities and clonal evolution, influencing the disease progression and treatment response [9, 10]. Beyond the conventional three-stage model, the new World Health Organization (WHO) classification attributes CML formation to a 2-stage regimen (the chronic phase and blast crisis). According to this model, the chronic phase is an early stage of the disease and the blast crisis is an advanced stage that is progressing. Increased phase as described in the classical model is regarded as one of the transitions between the two stages, instead of one of the sequential stages. The exclusion of the accelerated phase is also a result of the introduction of targeted tyrosine kinase inhibitors (TKI) treatment and disease surveillance, the latter of which has reduced the progression to advanced phases and rendered the accelerated phase less relevant. As a result, the present classification focuses on high-risk features and TKI-resistance [11].

This complex cascade of events underscores the need for a comprehensive understanding of CML pathogenesis for developing effective and precise therapeutic interventions. Understanding the structural aspects of BCR::ABL kinases is crucial in comprehending the complex structure of BCR::ABL proteins. The catalytic domain has a two-lobed shape, with an N-lobe and a C-lobe. The N-lobe mainly consists of β-sheets, while the C-lobe is characterized by alpha-helices [12]. A crucial element within this domain is the activation loop, particularly Thr315, a key residue involved in switching between two conformations: an active and inactive DFG motif, which is essential for catalysis [13].

There are several critical residues within the BCR::ABL catalytic domain, each playing a distinct role in its function. As previously stated, Thr315, also called the gatekeeper residue, is located in the ATP-binding pocket and can potentially influence inhibitor selectivity [14]. Asp363 residue, positioned in the catalytic loop, is responsible for phosphorylating the target substrate. The Tyr393 residue, found in the activation loop, serves as a phosphorylation target, regulating the kinase’s activity [13].

Most of the therapeutic strategies for CML focus on targeting the BCR::ABL protein with inhibitors. These inhibitors are mainly classified into two categories: DFG-in and DGF-out inhibitors attach to the BCR::ABL catalytic domain in its active form in the case of DFG-in and inactive form in the case of DFG-out. DFG-out inhibitors are particularly effective against variants of BCR::ABL resistant to DFG-in inhibitors [15].

The introduction of BCR::ABL inhibitors has drastically transformed the treatment approach for CML. These targeted therapies have substantially increased the survival rates of CML patients, transforming the condition from being fatal to a manageable state [16]. By inhibiting the BCR::ABL protein, these inhibitors prevent uncontrolled cell growth and proliferation, enabling patients to live healthier and longer lives.

### BCR-ABL protein isomers

**Fig. 1:**
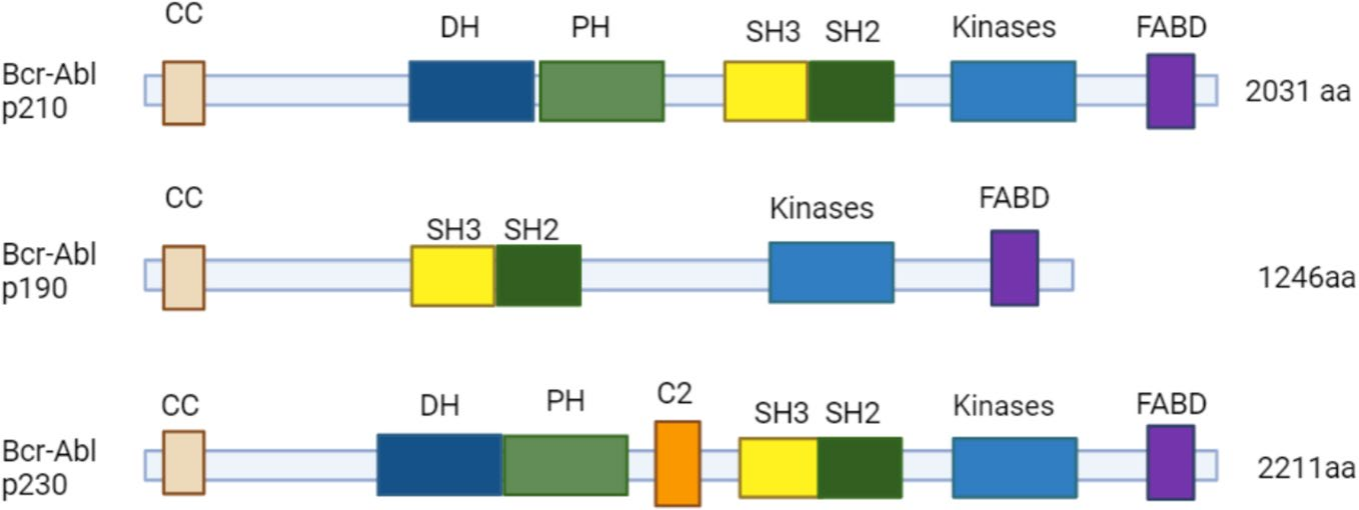
Major genotypic variations of BCR::ABL fusion gene, showing different domain compositions and their respective protein lengths (in amino acids)

The translocation of the BCR::ABL gene can give rise to four principal variants—P190, P185, P210, and P230, each capable of causing distinct forms of leukemia. The P210 BCR::ABL isomer is the most common form, found in approximately 95% of CML patients, where only 2.5% of them have BCR::ABL oncogene without Pleckstrin Homology (PH) domain [17]. The P190 BCR::ABL variant is linked to acute lymphoblastic leukemia (ALL) in 25% of adults and 5% of children [18, 19], while the P185 variant is associated with 15–20% of ALL. The P230 BCR::ABL isomer is linked to chronic myelomonocytic leukemia (CMML) [20]. BCR::ABL isoforms are the result of variant exon fusions during the translocation process. For example, P190 is derived from the fusion involving exon 1 of BCR, P210 from exon 13 or 14, and P230 from exon 19 (Fig. 1). These various fusion events result in various protein structures and, as a consequence, differences in disease phenotypes.

### Structure and pathophysiology of BCR::ABL P210 fusion proteins

The P210 BCR::ABL fusion protein consists of two domains, the BCR (Breakpoint Cluster Region) at N-terminal and the ABL (Abelson Murine Leukemia) kinase at C-terminal. The BCR subdomain contains an oligomerizing coiled-coil domain and a serine/threonine kinase domain [21]. The ABL region includes an SH2 (Src Homology 2) domain, SH3 (Src Homology 3) domain, kinase domain, and C-terminal actin-binding domain [22]. This fusion protein is trafficked to the cytoplasm resulting in its constant tyrosine kinase activity, which is essential for its oncogenic function.

The Dbl Homology (DH) domain causes the deregulation of the signaling pathways in CML. Additionally; the DH domain of the P210 variant regulates the activity of the protein. For example, the DH domain can be autophosphorylated which can either increase or decrease its activity [23].

**Fig. 2:**
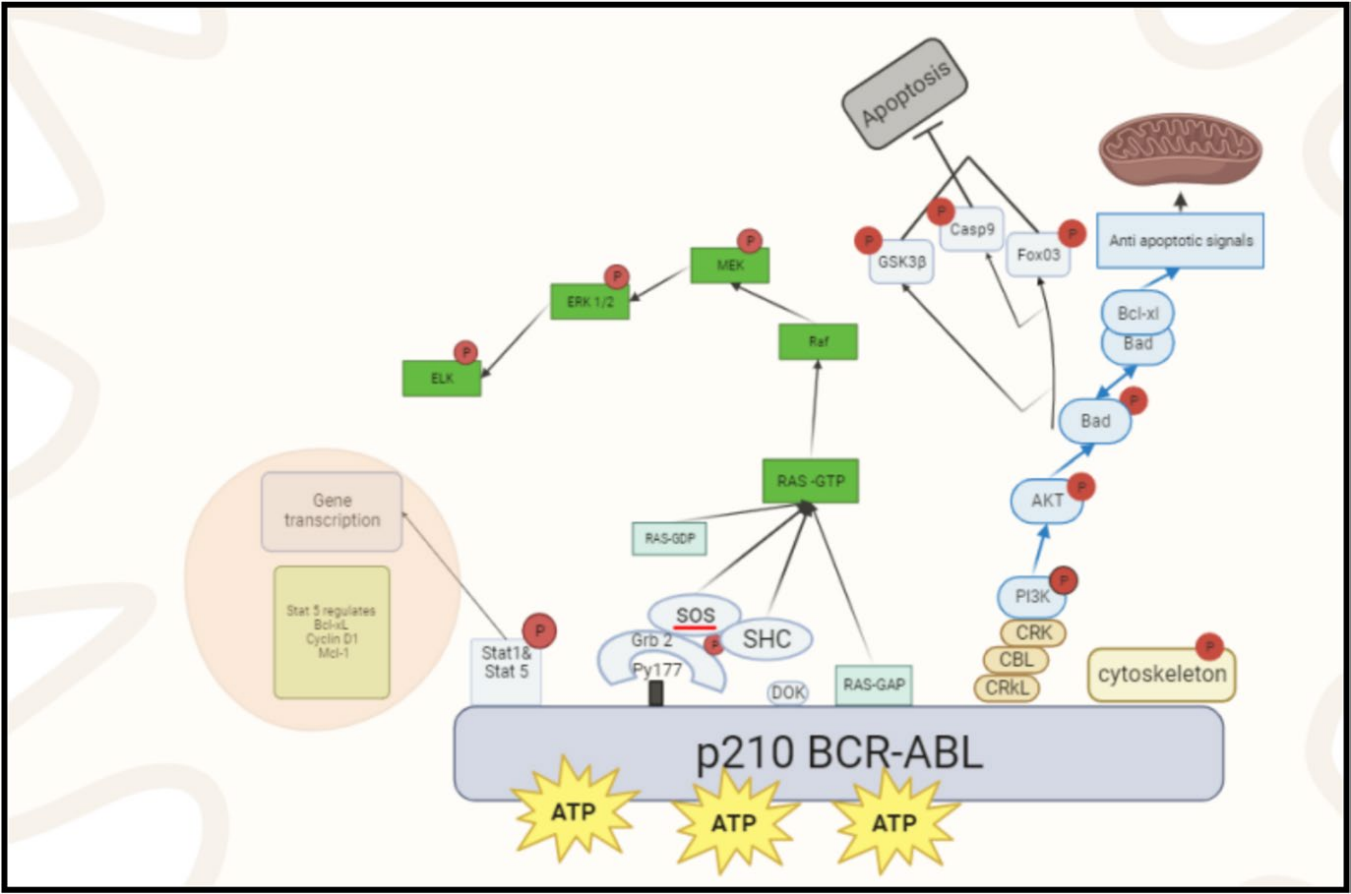
P210 isomer assisted BCR::ABL cascade-In the BCR::ABL cascade, p210 isomer stimulates downstream signaling pathways, such as Ras/MAPK and PI3K/Akt pathways, which promote cell proliferation and inhibit apoptosis. This dysregulated signaling cascade is a key driver of CML pathogenesis

The PH domain is involved in crucial events like binding to the phosphoinositides present in the inner membrane of cells (Fig. 2). This binding aids in the localization of the membrane, facilitating the emergence of downstream signaling partners and protein–protein interactions, where the PH domain interacts with a series of other proteins, including guanine nucleotide exchange factors, which play a crucial role in cellular signaling [24, 25].

The PH domain is also reported to bind to other proteins, such as proteins that contain SH2 domains like signaling proteins, adaptor proteins, and enzymes. The SH2 domain contains a pocket that can bind to a phosphotyrosine residue on a protein. The stabilization of the protein on the membrane and its close proximity to other signaling proteins can be facilitated by the interaction between the PH domain and the SH2 domain [26–28]. The PH domain can also be anchored to the membrane by a lipid anchor. The lipid anchor inserts into the membrane, anchoring the protein to the lipid bilayer.

Various factors influence the pathway for membrane localization of PH domain-containing proteins with protein–protein interaction (PPI), including the type of phosphoinositide (PI) and the type of protein with which the PH domain networks, in the presence of a lipid anchor. This pathway is crucial for the functioning of several distinct proteins, such as signaling proteins, cytoskeletal proteins, and membrane-associated enzymes [28].

### Pathophysiology of BCR::ABL P190 fusion proteins

**Fig. 3:**
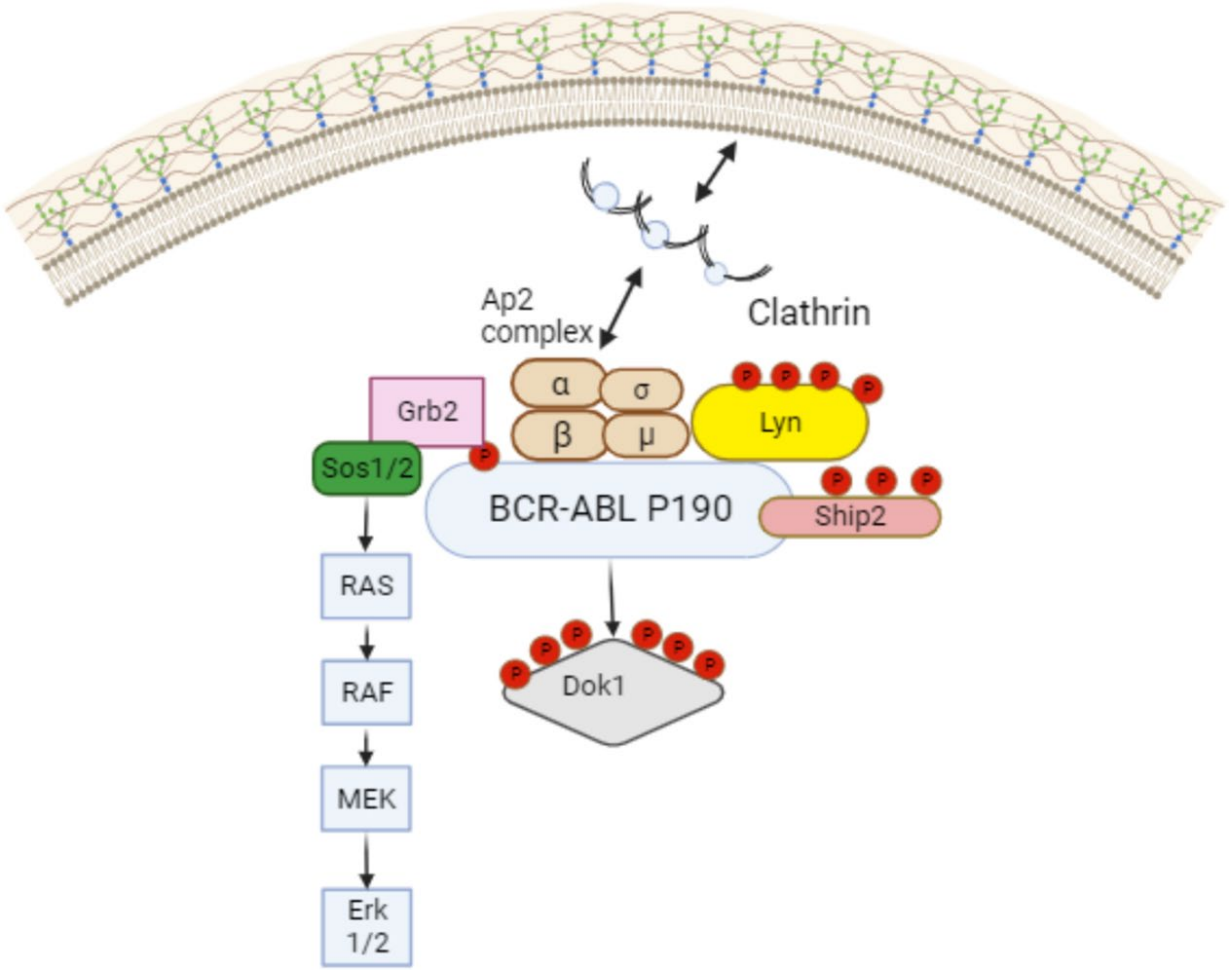
P190 BCR::ABL downregulation mechanism. Clathrin-mediated endocytosis, Lyn/Ship2/Grb2 binding, Dok1 recruitment, and Ras/RAF/MEK/Erk ½ pathway inhibiting BCR::ABL signalling

The P190 BCR::ABL protein, a shorter isoform of p210 BCR::ABL fusion protein, is 1246 amino acid long. However, it lacks portions of the PH and DH domains [29]. These missing segments result in functional alterations that influence the protein’s interactions and signaling capabilities. The PH domain plays a vital role in membrane localization. The absence of PH domain has disrupted the membrane localization process, which reduces the interactions with signaling partners altering pathways (Fig. 3). The DH acts as a regulatory element, and its truncation in P190 BCR::ABL may lead to altered kinase activity, potentially affecting the protein's ability to phosphorylate downstream substrates and regulate cellular signaling [29].

### P230 BCR::ABL: a distinct isoform with unique properties and resistance considerations

The P230 BCR-ABL kinase represents the rarest among the four primary isoforms of BCR::ABL, accounting for approximately 5% of chronic myeloid leukemia (CML) cases arising from an alternate breakpoint in the BCR gene. This variation leads to the formation of a larger fusion protein with additional BCR sequences [30]. Despite sharing the same BCR::ABL fusion, P230 BCR::ABL has the weakest transforming activity. This is because P230 BCR::ABL exhibits the lowest intrinsic tyrosine kinase activity and exhibits reduced phosphorylation of downstream signaling proteins compared to P210 and P190 BCR::ABL [22]. Resistance to TKIs may arise from C2 domain, a structural and functional regulator present in both P210 and P190 BCR::ABL variants. This domain may contribute to TKI resistance. Some CML patients resistant to TKIs have been found to harbor mutations in this C2 domain. Such mutations may alter the structure or function of the C2 domain, potentially affecting the protein interaction with TKIs or its ability to regulate BCR::ABL signaling. The C2 domain may also indirectly contribute to TKI resistance by modulating the protein localization and interaction with other proteins involved in TKI sensitivity [31]. The lower transforming activity of p230 BCR::ABL is reflected in its clinical presentation. CML patients with p230 BCR::ABL tend to have a more indolent course of the disease, with a lower incidence of blast crisis compared to patients with p210 or p190 BCR::ABL [32].

### Current treatments for CML conditions

TKIs are therapeutic drug targets against BCR::ABL proteins. They selectively bind to ATP-binding sites of BCR::ABL tyrosine kinase domain to induce apoptosis of TKI and prevent the transfer of phosphate group to tyrosine residues on substrate proteins. This inhibition of kinase activity disrupts the deregulated signaling pathways that drive CML, leading to apoptosis in CML cells and effective disease control [33].

TKI can further be classified into -

### First generation

Imatinib, first introduced in 1996, BCR::ABL inhibitor is a revolutionary therapeutic drug for CML. It remains the cornerstone for the chronic phase of CML [34]. Imatinib's mechanism of action aims to target the BCR::ABL protein kinase domain, effectively preventing its interaction with ATP, a critical molecule for cell division [35]. Imatinib has demonstrated remarkable efficacy in treating both the chronic and acute phases of CML [36], achieving long-term survival rates exceeding 90% [37]. However, the emergence of CML cases resistant to imatinib prompted the advancement of second-generation ATP-competitive inhibitors like nilotinib [38] and dasatinib [39], which exhibit enhanced potency and efficacy against certain imatinib-resistant patients.

### Second generation

Second-generation BCR::ABL inhibitors, including drugs like dasatinib, represent a pivotal advancement in chronic myeloid leukemia (CML) treatment strategies [40]. These inhibitors are more powerful and efficacious against the class of Bcr-Abl mutations than imatinib, in the first-line therapy for CML [41]. Their superior performance has led to their adoption as first-line therapy in recent years and as the treatment of choice for imatinib-resistant CML patients. The overall landscape of CML therapy has greatly improved due to second-generation inhibitors’ increased effectiveness and decreased toxicity, offering patients improved outcomes and expanded treatment options [40].

### Third generation

Third-generation BCR::ABL inhibitors ponatinib and asciminib are the most recent advances in CML therapy. These inhibitors have greater potency and a broader spectrum of activity against BCR::ABL mutations than first- or second-generation inhibitors [42–44]. Ponatinib is strongly active against the BCR::ABL fusion proteins having T315I mutation, which exhibits resistance to first and second-generation TKIs [45]. Asciminib, an allosteric inhibitor, has unique resistance mutation profiles and high cellular activity, which can provide a new strategy to prevent resistance when used in conjunction with next-generation TKIs [42].

Monobodies, single-domain proteins, show potential to be new therapeutic agents for CML because they are able to interact with BCR-ABL1 with high affinity. In vitro and ex vivo studies have demonstrated that monobodies display high inhibitory activity toward the BCR::ABL1 kinase, thus inducing the massive cell death of the CML cell lines [46]. Although monobody delivery has been achieved via lentiviral transduction or transfection into cell lines, in vivo delivery approaches are still to be developed. The development of safe and effective intracellular delivery technologies is of paramount importance for the clinical applicability of these promising molecules [47].

ATP-competitive inhibitors are usually tolerated well but may cause side effects like fatigue, nausea, diarrhea, and heart disease. Uncommon but serious adverse events such as liver dysfunction and pancreatitis have been described [48, 49].

### Targeting mechanism of imatinib

Imatinib suppresses the activation of the BCR::ABL tyrosine kinase domain by targeting its inactive conformation. This interaction stabilizes the inactive form of the BCR::ABL protein and inhibits it from phosphorylating downstream signaling proteins, such as STAT5 [15]. STAT5 is a transcription factor that controls the growth and survival of CML cells [50]. By inhibiting STAT5 activation, imatinib blocks the accelerated growth and progression of CML cells [51].

### Cellular effects of imatinib in CML

**Fig. 4:**
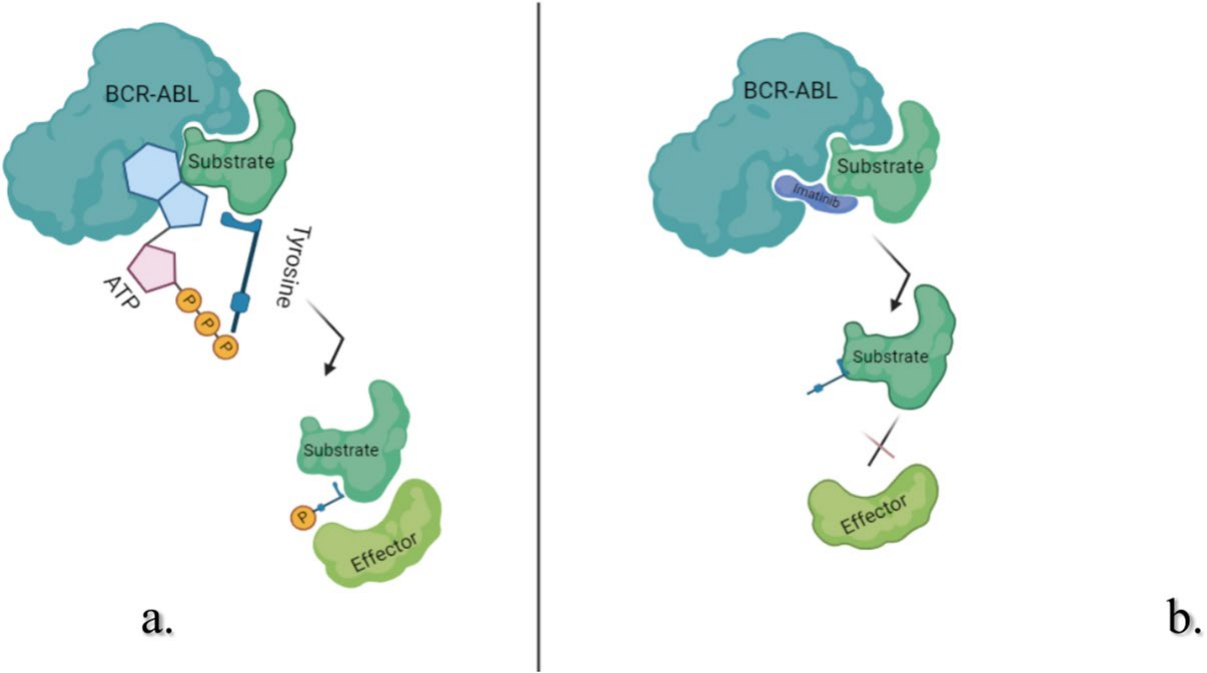
Imatinib mechanism of action. **a** BCR-ABL gene activating a signaling pathway by phosphorylating substrates, typically tyrosine residues, using ATP, enabling it to interact with effector molecules, triggering downstream cellular response. **b** Imatinib, after binding to BCR-ABL, inhibits the kinase activity and consequently, the substrate cannot be phosphorylated, disrupting the signaling cascade and preventing the activation of effectors

Imatinib inhibits the working of BCR::ABL tyrosine kinase, which primarily blocks pro-proliferative signaling pathways, ultimately leading to apoptosis [52]. Among these effects is the inhibition of CDK activation, preventing the CML cells from advancing through the cell cycle [53, 54]. CDKs enzymes regulate cell division [55]. Imatinib also induces cell death of CML cells by activating the caspase cascade (Fig. 4) [56]. During apoptosis, a class of enzymes called caspases disassembles the cellular components [57]. Imatinib can also induce differentiation of CML cells into mature myeloid cells a process, known as terminal differentiation, contributing to the long-term remission of the disease [58]. Furthermore, imatinib can increase the sensitivity of CML cells to other chemotherapy drugs, likely due to the inhibition of DNA repair pathways in these cells [59].

### Dasatinib—mechanism of action

Dasatinib acts on BCR::ABL down-regulation pathway and is effective against Imatinib-resistant CML [60]. Imatinib-resistant cells are reported to have reduced BCR::ABL gene expression, leading to activate pathways like Akt/mTOR/ PI3K [61]. The mTOR activation phosphorylates p70S6K, regulating apoptosis and promoting cell survival [62]. Dasatinib down-regulates Akt Ser473 phosphorylation [63], subsequently inhibiting the mTOR pathway and inducing apoptosis. Besides inducing apoptosis, Dasatinib has the ability to cause cell cycle arrest during various phases, which assists in inhibiting the growth of CML cells [64].

**Fig. 5:**
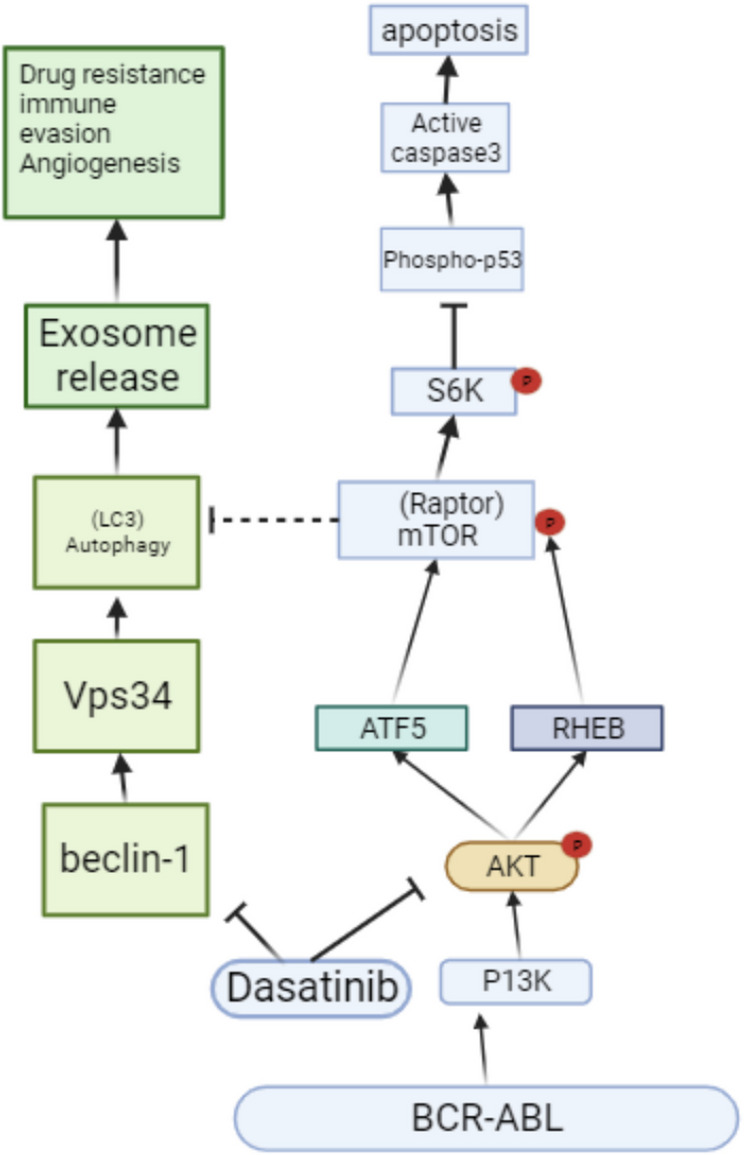
Dasatinib mechanism of action**—**Dasatinib’s dual effects on BCR::ABL and PI3K pathways can induce apoptosis via phosphor p53 and active caspase 3 while simultaneously promoting autophagy through beclin-1 and Vps34, influencing various cellular responses, including drug resistance and angiogenesis

Studies have reported that Dasatinib inhibits the autophagy pathway by downregulating beclin-1 and Vps34, ultimately leading to reduced exosome release (Fig. 5). Notch/Hes-1 signaling downregulates PTEN, leading to Akt activation and mTOR pathway induction [65]. Rapamycin and GSI, a type of Notch inhibitor, induce apoptosis by downregulating p-p70S6K [66]. Dasatinib synergistically enhances Rapamycin’s apoptosis-inducing effect. Additionally, the drug reduces the exosome release by suppressing the expression of beclin-1 and LC3-II [67]. It is essential to mention the clinical efficacy of Dasatinib in treating CML, including its role in achieving deep molecular responses and overcoming Imatinib resistance. Furthermore, Dasatinib exhibits an effective safety profile and tolerability, providing patients with a wide range of therapeutic options [68].

### Olverembatinib—mechanism of action

Olverembatinib specifically binds to and inhibits the FLT3 kinase domain. This binding inhibits the activity of FLT3, blocking its autophosphorylation and activation [69]. The inhibition of FLT3 disrupts downstream signaling pathways, like PI3K [70], MAPK, and STAT5 [71], which are accountable for abnormal cell growth and survival [72]. In particular, FLT3 mutations frequently co-occur with BCR::ABL gene in certain cases of acute myeloid leukemia (AML) [73]. The dual antagonistic activity of olverembatinib across those targets implicates the drug as a powerful treatment option for patients bearing both mutations, although the actual mechanistic interaction between FLT3 and BCR::ABL is still to be determined. In addition to its anti-proliferative behavior, olverembatinib has been proven to stimulate leukemic cell differentiation, resulting in decreased leukemic stem cell activity. This differentiation leads to its efficacy in killing leukemic cell populations and preventing disease relapse [74].

**Fig. 6:**
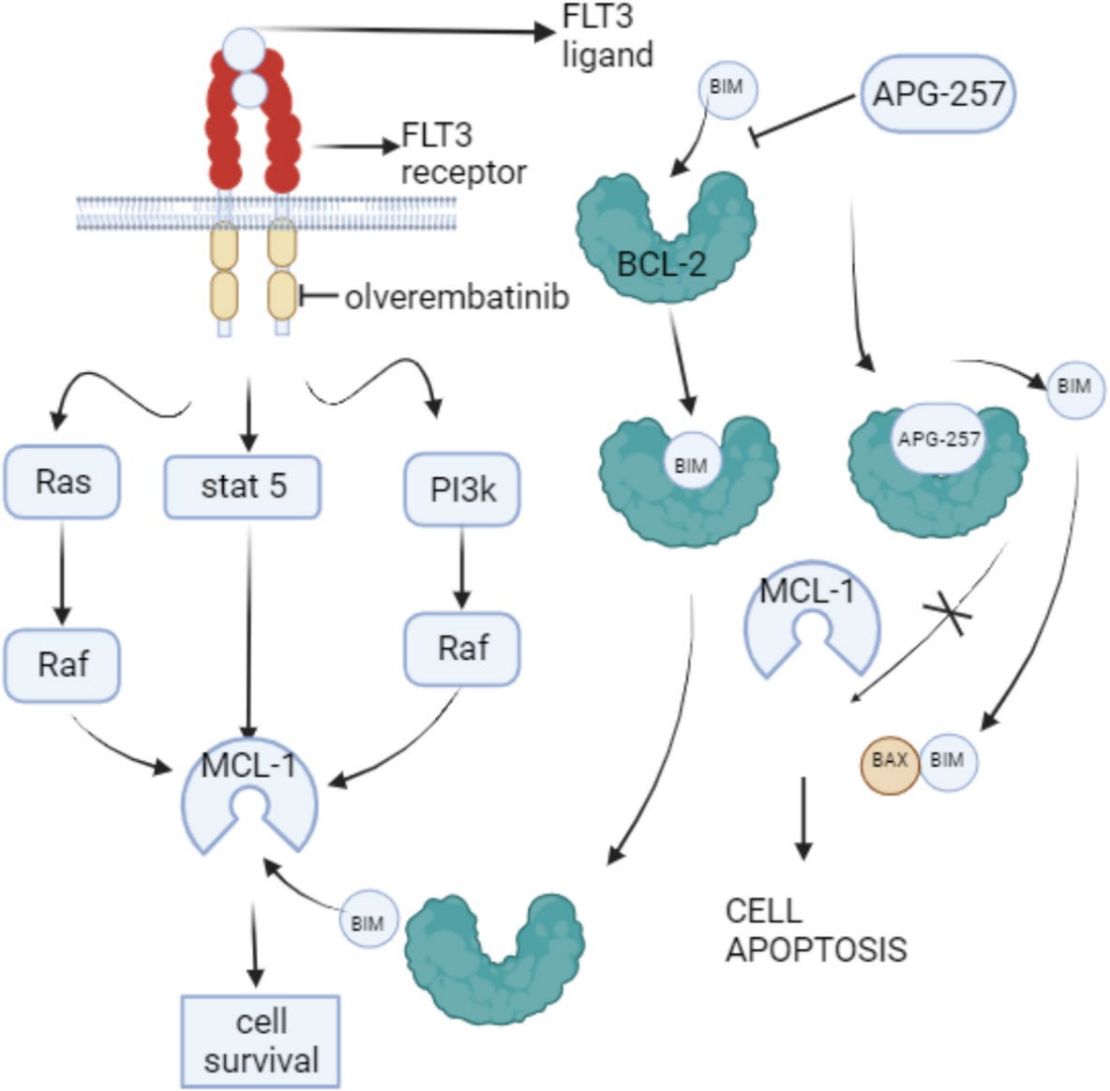
Olverembatinib mechanism of action—olverembatinib acts on FLT3 receptor, inhibiting its action and disrupting the downstream signaling pathway involving STAT5, Ras, and Raf, ultimately downregulating MCL-1 protein expression. It enhances the pro-apoptotic effects of BAX and BIM proteins while inhibiting BCL2 by the addition of APG-257, promoting cell apoptosis

Combination therapy with olverembatinib and a BCL-2 inhibitor, like APG-257, suppresses anti-apoptotic signals and promotes cell death (Fig. 6). This combination therapy can be achieved by downregulating MCL-1, leading to effective treatment of FLT3 mutant BCR-ABL gene cells which otherwise causes ALL [64]. It is important to note that olverembatinib currently is only approved in China and cannot be considered globally as the third-generation TKI. This regulatory status restricts its worldwide application but it points to its future approval potential in other countries [75].

### Asciminib—mechanism of action

Asciminib is a first-class allosteric inhibitor of BCR-ABL1, approved for the treatment of Philadelphia (Ph) chromosome-positive chronic myeloid leukemia (CML) in the chronic phase after prior exposure to two or more tyrosine kinase inhibitors (TKIs) [42]. Also, the superior effectiveness, safety, and tolerability of asciminib in newly diagnosed CML patients than conventional (ATP-competitive) TKIs are anticipated to lead to an extension of asciminib approval for first-line therapy [43].

Asciminib is a highly specific and potent inhibitor of BCR-ABL1 kinase activity and signaling. It exerts its effect through the binding to the allosteric myristoyl binding site of the ABL1 tyrosine kinase domain [76]. The major splice isoform 1b of ABL1 is myristoylated at its N-terminus. Intramolecular binding of the myristoyl moiety of its hydrophobic binding pocket in the kinase domain is a prerequisite for its anchoring to the SH3-SH2 domain clamp in the kinase domain. This crosstalk provides tight control over autoregulation of the proto-oncogene ABL1 most of the time in cells [77, 78]. As the Ph chromosome translocation replaces the first exon of ABL1, including the myristoylation site, with BCR sequences, the myristoyl binding pocket in the kinase domain lacks its endogenous ligand. This leads to kinase activation, constitutive signaling, and BCR-ABL1 oncogenicity [79].

**Fig. 7:**
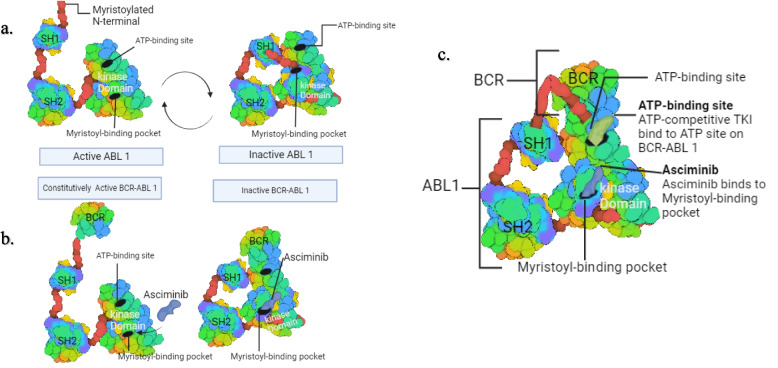
**a** In healthy cells, the ABL kinase activity is tightly regulated by the binding of the myristoylated N-terminal to the myristoyl binding pocket, maintaining the kinase in an inactive state. **b** In CML, this autoregulatory mechanism is disrupted by the formation of the BCR::ABL1 fusion oncoprotein, resulting in constitutive ABL1 kinase activation. Asciminib exerts its inhibitory effect by specifically targeting the myristoyl-binding pocket, restoring the inactive form and effectively suppressing ABL1 kinase activity. **c **Asciminib stands apart from ATP-competitive tyrosine kinase inhibitors (TKIs) by virtue of its unique mode of action, targeting the ABL myristoyl pocket (STAMP) in place of competing with ATP binding site

Asciminib binds to the myristoyl pocket, triggering a conformational change in the C-terminal α-helix of the kinase domain (α-I’-helix). This allows SH3-SH2 domain clamp binding to the kinase domain, leading to inhibition of BCR-ABL1 (Fig. 7). Therefore, the functional integrity of both SH3 and SH2 domains is indispensable for BCR-ABL1 inhibition by asciminib [80].

Asciminib has shown the effect of synergy when used with other targeted agents or with conventional chemotherapeutic agents. For instance, combination therapy utilizing ponatinib, another effective BCR-ABL1 inhibitor, has achieved a better performance in CML models by overcoming resistance and decreasing the disease burden. These synergistic effects may also suppress the development of resistance [81, 82].

It is known that asciminib treatment, even in the context of heavily pretreated CML resistant to several previous TKIs, is able to achieve deep and long-lasting responses. These long-term molecular responses are coupled with improved progression-free survival and better quality of life, positioning asciminib as a potential salvage treatment [51, 83, 84].

### Reasons for resistance

While imatinib was pioneering in CML therapy, point mutations in BCR::ABL kinase domain, such as T315I, E255K/V, and Y253H, alter the conformation of the kinase, leading to reduced effectiveness and decreased binding affinity [85–89]. Overexpression of drug efflux pumps (e.g., ABC transporters such as P-glycoprotein) [90] and the clonal evolution of CML cells with genetic alteration may confer resistance to imatinib. Other potential reasons include pharmacokinetic factors such as poor absorption, or incomplete inhibition, as well as BCR::ABL gene amplification, all of which can reduce the drug’s therapeutic efficacy [91].

Similar to imatinib, the emergence of mutants in BCR::ABL gene (e.g., T315I, F317L) can render Dasatinib less effective by diminishing its binding affinity to the target. The efficiency of Dasatinib may also decline due to efflux pumps, such as P-glycoprotein, which reduces the concentration of the drug within the cells [92]. Enhanced protein degradation of BCR::ABL via the ubiquitination-proteosome system leads to reduced inhibition of leukemic cell survival pathways, thereby serving as a mechanism of dasatinib resistance [93]. Moreover, resistance could also arise due to other factors such as activation of alternative signaling pathways (e.g., PI3K/AKT, JAK/STAT), metabolic reprogramming of leukemic cells, and changes in cellular metabolism.

To overcome this resistance, third-generation drugs like olverembatinib and asciminib were designed to structurally inhibit a wide spectrum of BCR::ABL mutants. Olverembatinib specifically targets the T315I mutation, while asciminib binds to the allosteric sites of BCR::ABL domain kinase. Ultimately, both of these drugs have demonstrated cumulative efficacy against resistant CML cells individually, instilling new hope to overcome therapeutic barriers. Overall inhibition profiles of TKIs are provided in Table 1.

**Table 1:** Inhibition profile of BCR-ABL kinase inhibitors

Drug	Imatinib	Dasatinib	Nilotinib	Bosutinib	Ponatinib	Olverembatinib	Asciminib
TKI generation	First	Second	Second	Second	Third	Third	Third
Level of inhibition	Moderate to high inhibition	High inhibition, including some resistant mutants (E255K/V, F317, LY253H), excluding T315I	High inhibition, effective against some resistant mutants	Moderate to high inhibition	High inhibition, effective against T315I	High inhibition, designed to overcome resistance	High inhibition, particularly effective against resistant mutants
Mode/mechanism of targeting	At the ATP-binding pocket within the catalytic domain of BCR::ABL kinase	Binds to multiple conformations of the kinase domain, primarily to ATP-binding pocket	Binds to inactive conformation of BCR::ABL1 kinase (ATP-binding pocket)	Inhibits both active and inactive forms of BCR::ABL1	Inhibits BCR::ABL1, including the T315I mutation	Binds firmly to *the ATP-binding site* of both native BCR::ABL1 and various BCR::ABL1 mutants (Q252H, E255K, F317L, F317I M351T, H396P)	Selectively binds to the allosteric site, exactly at the myristoyl binding site
Binding site/residue	Tryptophan 261 (Trp261) Tyrosine 253 (Tyr253) Glutamic Acid 286 (Glu286) Threonine 315 (Thr315)	Threonine 315 (Thr315) Phenylalanine 317 (Phe317) Tyrosine 253 (Tyr253) Threonine 315 (Thr315)	Phenylalanine 317 (Phe317), ATP-binding residues	Tyrosine 253 (Tyr253) and others in ATP-binding pocket	Threonine 315 (Thr315), ATP-binding site residues	Specific residue may vary depending on the unique binding site targeted by the drug, within the kinase domain of BCR::ABL	Binding to the allosteric site of BCR::ABL, away from the active site (specific residue not disclosed publicly)
Efficiency against BCR::ABL variant	p190—low to Moderate p210—high p230—moderate to high	High (except for T135I)	High (in all 3 variants)	Moderate to High (in all 3 variants)	High (in all 3 variants)	High (in all 3 variants)	High (in all 3 variants)
Dosage level	400 mg daily [80]	70–100 mg QD daily [81]	300–400 mg twice daily	500 mg daily	45 mg daily	30–50 mg daily [69]	40–200 mg BID [82]
Possible cross-reactions	-Generally, well-tolerated -May interact with drugs that affect CYP3A4 metabolism	-Similar to Imatinib, may interact with drugs metabolized by CYP3A4	May interact with CYP3A4 substrates	May interact with CYP3A4 substrates	May interact with CYP3A4 substrates	Not extensively reported	Not extensively reported
Side effects	Thrombocytopenia, diarrhea, nausea, muscle cramps, edema, fatigue, rashes	Fluid retention, headache, pleural effusions, nausea, diarrhea, myelosuppression fatigue; occasionally pulmonary hypertension and muscle aches	Myelosuppression, elevated liver enzymes, rash	Diarrhea, nausea, rash, liver enzyme elevations	Hypertension, vascular occlusion, myelosuppression	Skin hyperpigmentation, severe thrombocytopenia, hyperuricemia, hypertriglyceridemia, proteinuria, anemia, leukopenia, neutropenia	Fatigue, anemia, neutropenia, thrombocytopenia, pancreatitis, hypertension, arthralgias, nausea
Key mutations inducing resistance	M351T, E255K/V, V299, G250E, Y453	T315I, F317I, V299	Y253H, E255K, F317L	E255K/V, F317L, T315I	T315I	Most refractory gatekeeper mutant- T315I, and Q252H, E255K, F317L, F317I, M351T, H396P	T315I/ Y253H, other mutations near myristoyl-binding pocket include-A337V, P465S, I502, and V468F

### Preclinically validated inhibitors

#### Emerging therapeutic options for CML

Despite the remarkable advancements in TKIs, some CML patients become resistant to these medications. This resistance often stems from mutants at the BCR::ABL1 kinase domain (KD), the region that interacts with TKIs. Next-generation sequencing (NGS) is a potent technique to detect these mutations with high sensitivity and accuracy.

In response to these challenges, several promising third-generation TKIs have demonstrated high efficacy in preclinical studies of CML treatment. These TKIs, including bafetinib (INNO-406) [94], rebastinib (DCC-2036) [95], tozasertib (MK-0457, VX-680) [96], and HG-7–85-01 [97], hold the potential to overcome the limitations of existing TKIs and offer CML patients more durable and efficient therapy.

#### Bafetinib (INNO-406)

Bafetinib exhibits an inhibitory effect against both BCR::ABL and Lyn tyrosine kinases and is reported to influence the development and progression of CML [97]. Studies have proven that bafetinib has higher efficacy than imatinib, with fewer associated side effects [98]. As the drug attaches itself to the ATP-binding site of BCR::ABL kinase, it exerts its therapeutic activity by effectively blocking the kinase domain’s ability to transfer phosphate groups to target proteins. This restriction of BCR::ABL kinase activity leads to a reduced progression and survival of CML cells.

#### Rebastinib (DCC-2036)

Rebastinib is a BCR::ABL-selective inhibitor that exhibits excellent activity against TKI-resistant BCR::ABL1 mutations [99]. Preclinical models of TKI-resistant CML have demonstrated improved efficacy of rebastinib, which is currently undergoing clinical trials to evaluate its effectiveness in treating patients with TKI-resistant CML [96].

#### Tozasertib (MK-0457, VX-680)

Tozasertib is a dual inhibitor of BCR::ABL and c-SRC tyrosine kinases. The drug has proven efficacy against a wide range of BCR::ABL1 mutations, especially against TKI-resistant mutants. Clinical studies are being conducted on tozasertib to assess its effectiveness in treating CML [96, 100].

#### HG-7–85-01

HG-7–85-01 is a TKI with promising preclinical activity against CML [101]. Being a type-II ATP competitive inhibitor that binds to the ATP-binding site of the BCR::ABL kinase domain, it effectively prevents the transfer of the phosphate group to its target proteins. This inhibition leads to a decline in the progression and persistence of CML cells.

HG-7–85-01 has promising action against a wide class of BCR::ABL mutations, resistant to other TKIs [102, 103]. Preclinical models of TKI-resistant CML have also shown its efficacy. Clinical trials for HG-7–85-01 are presently being conducted to evaluate its effectiveness in treating patients with CML. Early results of trials have been encouraging, demonstrating the effectiveness and tolerability of HG-7–85-01 in CML patients [102, 103].

These emerging TKIs represent promising therapeutic avenues for CML, offering the potential to combat TKI resistance and offer highly effective and durable treatment options for individuals with this condition.

## Conclusion

The development of TKIs has made great strides in treating chronic CML, transforming it from a fatal condition to a manageable one. TKIs specifically target the BCR::ABL gene, the main driver for CML, by inhibiting its aberrant tyrosine kinase activity. Structural studies play a vital role in understanding the interplay between TKIs and BCR::ABL, providing crucial insights into their binding modes, selectivity, and potency. These insights have guided the rational design of novel and more effective TKIs, significantly improving the treatment landscape for CML patients.

## Data Availability

No datasets were generated or analysed during the current study.
